# 

**Published:** 1907-11-23

**Authors:** 


### A MODERN MEDICAL JOURNAL.

A new series of The Hospital was commenced with the weekly issue dated April 6, 1907. Arrangements were then completed to publish The Hospital as the modern medical journal, appearing weekly, price 3d., and containing not more than thirty-two pages of literary matter, mainly printed in long primer type, so as to form comfortable reading for the doctor in his carriage.

The Editorial Staff.

As its name implies, the new series of The Hospital will include every section of hospital work. A number of representative members of the medical profession, including some of the most active workers and best writers, now constitute the staff. All sections of medical and surgical work are included in the paper, each section being under the management of an editor selected because of his association with, and knowledge of, the subjects comprised in it.

Its Objects.

Care will be taken to include in the paper every modern development and all new discoveries, together with the methods of treatment and practice in this country, in the great European schools of medicine, and in the United States. The object will be to endeavour to make each section of the paper both compact and complete; and the whole will be written with the desire to interest, help and keep up to date practitioners engaged in the active service of their profession.

A Clinic Every Week.

A Clinical Lecture by some leading member of the medical profession and addressed to graduates is published each week. Thus the earlier numbers have contained clinics on "The Treatment of Acute Pneumonia," by Sir Dyce Duckworth, M.D., LL.D., F.R.C.P.; on "Stiffness of the Spine," by Sir William Bennett, K.C.V.O.; on "Unsoundness of Mind not always Insanity," by G. H. Savage, Esq., M.D., F.R.C.P.; on "The Modern Treatment of Syphilis," by D'Arcy Power, Esq., M.A., M.B., F.R.C.S.; on "Operations on the Bile Passages," by G. H. Makins, Esq., C.B., F.R.C.S.; and on "Common Forms of Dyspepsia in Women," by Robert Saundby, Esq., M.D., F.R.C.P.

Writers to Come.

The following, amongst others, have agreed to contribute Clinics and other articles during the ensuing year:—

**A Clinical Lecture by Wm. Osler, M.D.Oxon., F.R.C.P., F.R.S., LL.D., Regius Professor of Medicine, University of Oxford.**

**A Clinical Lecture by Sir J. Halliday Croom, M.D.Edin., F.R.C.P., F.R.C.S., Consulting Gynæcologist to the Edinburgh Royal Infirmary; Physician Edinburgh Royal Maternity Hospital; Professor of Midwifery at the Edinburgh University.**

**On Syphilitic Cachexia. By Sir Dyce Duckworth, M.D., LL.D., F.R.C.P., Consulting Physician St. Bartholomew's Hospital; Senior Physician Seamen's Hospital, Greenwich.**

**On Waters and Climate. By J. F. Goodhart, M.D., C.M., F.R.C.P., LL.D., Consulting Physician Guy's Hospital.**

**A Clinical Lecture. By Robert Saundby, M.D.Edin., C.M., F.R.C.P., Professor of Medicine Birmingham University; Physician Birmingham General Hospital.**

**A Clinical Lecture. By William Hale White, M.D., F.R.C.S., Physician and Lecturer on Medicine Guy's Hospital.**

**A Clinical Lecture. By H. Gilbert Barling, F.R.C.S.Eng., Professor of Surgery and Ingleby Lecturer University of Birmingham; Surgeon Birmingham General Hospital.**

**On Oral Sepsis in its Relation to Abdominal Disease. By J. H. Dauber, M.N., B.Ch.Oxon., M.A., Gynæcologist Hospital for Women, Soho.**

**On the Importance of Localising Heart Beats. By Arthur Foxwell, M.A., M.D.Cantab., F.R.C.P., Professor of Therapeutics Birmingham University; Physician Queen's Hospital, Birmingham.**

**A Clinical Lecture. By Sir R. Douglas Powell, K.C.V.O., M.D., F.R.C.P., Physician Extraordinary to the King; Consulting Physician, Middlesex and Brompton Hospitals.**

**On Syphilitic Diseases of the Joints. By D'Arcy Power, M.A., M.B., F.R.C.S.Eng.; Surgeon and Lecturer on Surgery, St. Bartholomew's Hospital.**

**A Clinical Lecture. By P. H. Pye-Smith, M.D., F.R.C.P., Consulting Physician, Guy's Hospital.**

**A Clinical Lecture. By R. W. Philip, M.A.Edin., M.D., F.R.C.P., Lecturer on Practical Physic, Clinical Medicine, and Diseases of the Chest, Edinburgh School of Medicine; Examiner in Clinical Medicine, University and Royal College of Physicians, Edinburgh; Physician, Edinburgh Royal Infirmary.**

**On The Really Useful in Electro-Therapeutics. By H. Lewis Jones, M.A., M.D., F.R.C.P., Medical Officer in Charge of Electrical Department, St. Bartholomew's Hospital.**

**A Clinical Lecture. By C. R. B. Keetley, F.R.C.S., Senior Surgeon, West London Hospital.**

**A Clinical Lecture. By Jordan Lloyd, M.D., M.S., F.R.C.S., Surgeon, Queen's Hospital, Birmingham; Examiner in Surgery, Durham University.**

**A Clinical Lecture. By Sir George Beatson, K.C.B., M.D., C.M., Examiner in Surgery, Edinburgh University; Visiting Surgeon, Glasgow Western Infirmary; Surgeon, Glasgow Cancer Hospital.**

**A Clinical Lecture. By Charters J. Symonds, M.D., M.S. F.R.C.S., Examiner in Surgery, London University Surgeon and Lecturer on Surgery, Guy's Hospital.**

**On Some Medico-Legal Reminiscences. By A. P. Luff, M.D., F.R.C.P., D.Ph., Lecturer on Medical Jurisprudence and Toxicology, St. Mary's Hospital; Physician, St. Mary's Hospital.**

**On Some Medical Hæmorrhages. By Leonard Williams, M.D., M.R.C.P., Physician, French Hospital.**

**On Certain Urinal Diseases. By Reginald Harrison, F.R.C.S., Consulting Surgeon, St. Peter's Hospital for Stone.**

The General Practitioner.

There will be a General Practitioners' Page, and to this contributions are invited from practitioners upon actual experiences in the management of cases and in the treatment and diagnosis of disease.

"The Relaxations of a Medical Man" has proved to be a subject which is helpful and interesting in many ways to many active workers in the profession.

The Hospital is successfully providing for the practitioner an up-to-date modern medical journal, which supplies him each week with information and facts of immediate value in the conduct of his practice and in the discharge of the manifold duties which fall to his lot in the course of each day's work.

### THE GRADUATES' MEDICAL SCHOOLS.

### Diary for the week November 25 to 30.

**ROYAL SOCIETY OF MEDICINE, THERAPEUTICAL AND PHARMACOLOGICAL SECTION, Apothecaries' Hall, Blackfriars.**

**At 4.30 p.m.**—November 26, Mr. James Cantlie, **Some Tropical Diseases and the Remedies required for their Treatment or Prophylaxis.**

Dr. William Murray, **Therapeutics of Indigestion.**

**MEDICAL GRADUATES' COLLEGE AND POLYCLINIC, Chenies Street, W.C.**

**Daily** (except Saturday): Clinics, Demonstrations and Practical classes.

**Lectures at 5.15 p.m.**

November 25, Mr. Vernon Coghill, **Corneal Ulcers.**

November 26, Dr. M. C. Hawkes, **Swedish Medical Gymnastics in Spinal Curvature.**

November 27, Dr. W. Milligan, **Treatment of Suppuration in the Nasal Sinuses.**

November 28, Dr. Peter Horrocks, **Profuse Menstruation.**

**THE POST-GRADUATE COLLEGE, West London Hospital, Hammersmith, W.**

**Daily at 2:** Medical and Surgical Clinics; **at 2.30:** Operations.

**Special Departments at 10** (Tuesday, Wednesday, Friday), and daily at 2.

**Lectures at 5 p.m.**

November 25, Dr. Ball, **Nasal Suppuration** (I.).

November 26, Dr. Ball, **Nasal Suppuration** (II.).

November 27, Dr. Beddard, **Practical Medicine** (V.).

November 28, Mr. Edwards, **Practical Surgery.**

November 29, Dr. Drummond Robinson, **Gynæcology.**

**WEST END HOSPITAL FOR DISEASES OF THE NERVOUS SYSTEM, Welbeck Street, W.**

November 28, Dr. F. S. Palmer, **The Differential Diagnosis of Some of the more Common Types of Paralysis.**

**LONDON SCHOOL OF CLINICAL MEDICINE, Seamen's Hospital, Greenwich.**

**Medical and Surgical Clinics.**—Demonstrations and special departments daily.

**Lectures.**

November 26, **2.15 p.m.**, Dr. Hewlett, **The Use of Sera and Vaccines in Medical Treatment.**

November 28, **3.15 p.m.**, Sir Wm. Bennett, **Dilatation of the Stomach.**

**NORTH-EAST LONDON POST-GRADUATE COLLEGE, Prince of Wales's Hospital, Tottenham, N.**

**Surgical, Medical and Special Clinics** daily, Saturdays excepted.

**Special Lectures and Demonstrations.**

November 26, Dr. Chappel.

November 28, Dr. A. E. Giles, **Ultimate Results of Inoperable Gynæcological Cases.**

**NATIONAL HOSPITAL FOR THE PARALYSED AND EPILEPTIC, Queen's Square, Bloomsbury, W.C.**

**Lectures at 3.30 p.m.**

November 26, Dr. Ormerod, **Locomotor Ataxy.**

November 29, Mr. Ballance, **Surgery of the Nervous System.**

### BOOKS RECEIVED.

J. WRIGHT AND CO., BRISTOL.

"The Re-Education of Co-ordination by Movements." With chart. By A. G. Dampier-Bennett, M.R.C.S.

"Physical Methods in the Treatment of Heart Disease." By A. G. Dampier-Bennett, M.R.C.S.

"Pelvic Inflammations in the Female." By Thos. Wilson, M.D.

FUNK AND WAGNALLS CO.

"The Standard Family Physician." 3 vols.

PHARMACEUTICAL SOCIETY.

"The British Pharmaceutical Codex," 1907.

CHURCH OF ENGLAND TEMPERANCE SOCIETY.

"Sidelights on Alcohol." By "Medicus Abstinens."

LONGMANS AND CO.

"Text-book of Organic Chemistry for Medical Students." By G. v. Bunge. Translated by R. H. Aders Plummer, D.Sc.

"The Reduction of Cancer." By the Hon. Rolls Russell.

